# Data characterizing a panel of biodegradable cross-linked polyester implants for sustained delivery of an anti-viral drug

**DOI:** 10.1016/j.dib.2024.111182

**Published:** 2024-12-04

**Authors:** Sungmin Jung, Jack Bufton, Zeqing Bao, Wonjoon Cho, Dean Aguiar, Christine Allen

**Affiliations:** a Leslie Dan Faculty of Pharmacy, University of Toronto, Toronto M5S 3M2, Canada; b Pendant Inc, JLabs Toronto, 661 University Avenue, Suite 1300, Toronto M5G 0B7, Canada; c Department of Chemical Engineering & Applied Chemistry, University of Toronto, Toronto, ON M5S 3E5, Canada

**Keywords:** Pharmaceutics, Long-acting therapeutics, Polymers, Tenofovir alafenamide, Sustained release

## Abstract

Tenofovir alafenamide (TAF) is currently administered orally to patients for treatment of chronic hepatitis B virus infection and as a part of a combination therapy for human immunodeficiency virus (HIV) infection. A long-acting delivery system could provide several advantages as a formulation strategy for this drug including improved patient adherence, convenience, more consistent drug levels and potentially fewer side effects. To date, the vast majority of polymer-based long-acting delivery systems have been prepared from poly(lactide-*co*-glycolide) [1]. To expand the range of polymers available for use, cross-linkable allyl functionalized, polyester copolymers were considered for preparation of disc-shaped, implantable delivery systems for TAF. The physico-chemical properties of the implants were evaluated including thermal and spectral properties as well as *in vitro* stability. Subsequently the discs were loaded with TAF via a swelling-equilibrium approach and the discs were further characterized including, TAF loading and *in vitro* drug release. This dataset shows the potential of using these polymeric materials as a sustained delivery platform for TAF. The dataset is freely available on Mendeley Data.

Specifications Table

**Table:** 

Subject	Materials Application
Specific subject area	Evaluating the physico-chemical properties and release kinetics of tenofovir alafenamide loaded cross-linked polyester implants
Type of data	Tables Figures
Data collection	^1^H NMR spectra were recorded using a Bruker AMX 400 spectrometer. The molecular weight of the polymers was evaluated using an Agilent gel permeation chromatography (GPC) system equipped with a 1260 Infinity isocratic Pump and a refractive index detector. Two Agilent columns (PLgel mixed-D and mixed-C) were used with dimethylformamide (DMF) as the mobile phase at a flow rate of 1.0 mL/min. Thermal properties of the polymers as received, and the cross-linked discs were measured with a TA Instruments Q100 differential scanning calorimeter (DSC). Thermogravimetric analysis (TGA) measurements on the polymers as received and the cross-linked discs were carried out using a TA instruments Q50 analyzer. Fourier transform infrared spectroscopy (FT-IR) spectra of all samples were recorded using a Nicolet FT-IR spectrometer attached to an attenuated total reflectance (ATR) accessory. *In vitro* release of TAF from the discs was measured using an Agilent 1200 High-Performance Liquid Chromatography (HPLC) system coupled with a diode-array detector (DAD) for drug analysis.
Data source location	Leslie Dan Faculty of Pharmacy, University of Toronto 144 College Street, Ontario, M5S 3M2, Canada
Data accessibility	Repository name: Mendeley Data Data identification number: 10.17632/jmd69fbd94.1 Direct URL to data: https://data.mendeley.com/datasets/jmd69fbd94/1
Related research article	J. Bufton, S. Jung, J. Evans, Z. Bao, D. Aguiar, C. Allen. Cross-linked valerolactone copolymer implants with tailorable biodegradation, loading and *in vitro* release of paclitaxel. European Journal of Pharmaceutical Sciences. 162 (2021) 105,808. 10.1016/j.ejps.2021.105808

## 1 Value of the Data

• Drug Delivery Innovation: The data demonstrates the formulation of TAF-loaded disc-shaped implants using a cross-linkable polyester-based material. This highlights that alternatives to poly(lactide-*co*-glycolide) can be used to develop long-acting delivery systems.
• Drug Release Optimization: The study on varying copolymer compositions reveals how adjustments can control the implants' physico-chemical properties and TAF release rates. This finding supports the customization of drug delivery systems to meet therapeutic requirements, advancing personalized medicine in chronic viral infection treatments.
• Clinical Need Fulfillment: This research highlights the development of a sustained-release system for TAF, addressing the need for less frequent dosing in antiviral therapies. It contributes to pharmaceutical sciences by offering a new approach to improve treatment outcomes.

## 2 Background

Tenofovir alafenamide (TAF), a drug that is used to treat several viral infections (*i.e.*, HIV-1, HIV-2, and hepatitis B) has a relatively short biological half-life. Long-acting anti-viral therapeutics offer several advantages over oral dosage forms and are a particularly attractive strategy for populations with low medical access–and–infrastructure [2]. A number of implants prepared from various materials including silicone [3], polycaprolactone [4,5], polyurethane [6] and titanium [7] and have been evaluated for long-acting TAF delivery preclinically. Non-biodegradable implants (*e.g.*, silicone or titanium) require eventual removal increasing the barrier to clinical adoption while biodegradable implants (*i.e.*, polyurethane) have shown increased local inflammation and necrosis compared to placebo *in vivo* [6,8]. Thus, there is a need to explore alternative biodegradable materials for sustained TAF delivery.

In this context, a panel of aliphatic polyester copolymers functionalized with pendant allyl groups were used to prepare cross-linked disc implants for sustained delivery of TAF. To identify the optimal formulation for TAF delivery, discs were prepared from four different cross-linked copolymers. The findings revealed that the specific choice of polymer composition significantly affects the implants' degradation rates *in vitro*, the efficiency of TAF incorporation, and the control over its release. These insights confirm that adjusting polymer composition offers a strategic approach to customizing the delivery system's characteristics to meet targeted therapeutic needs.

## 3 Data Description

**Fig. 1:**
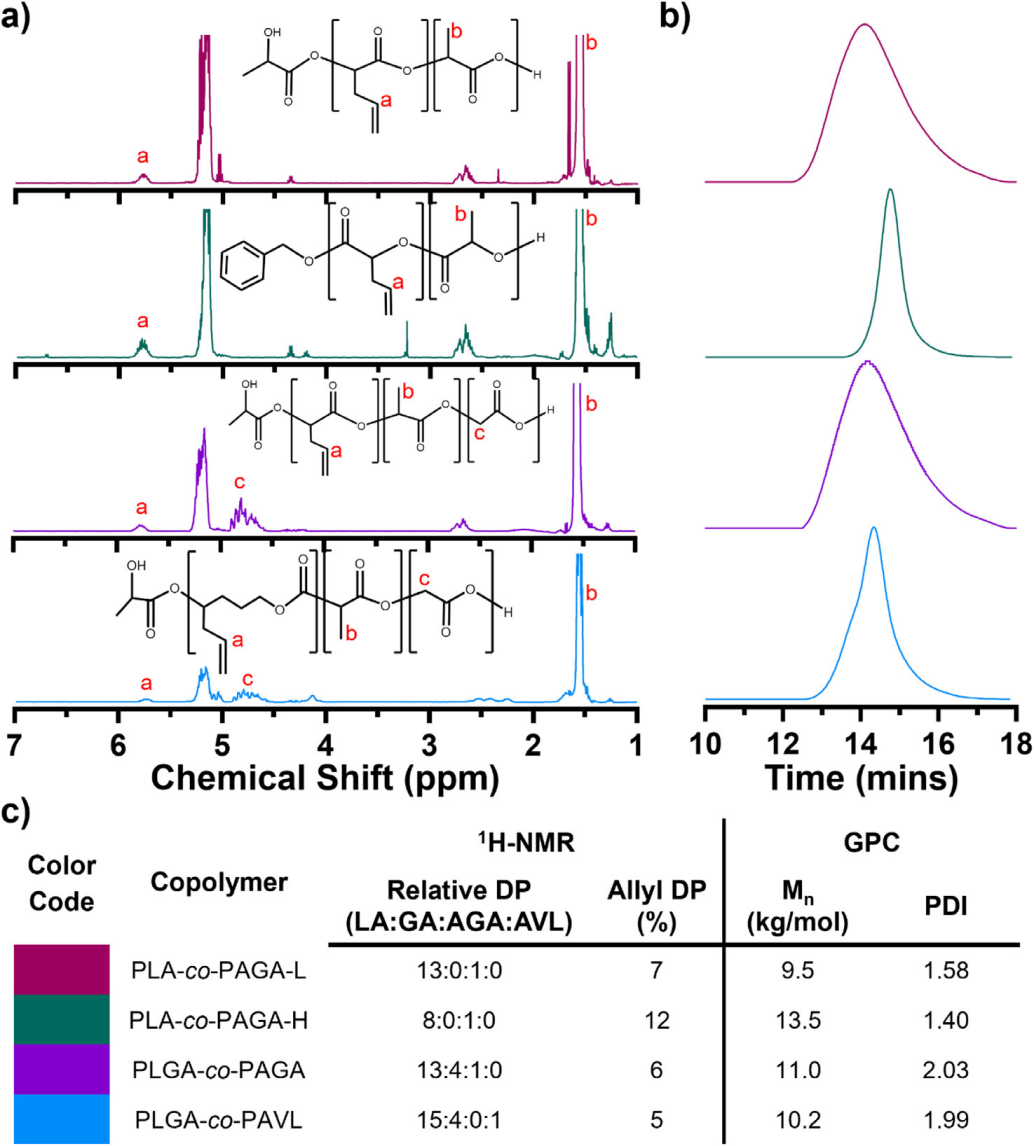
**a)**^1^H NMR spectra and **b)** GPC traces of poly(lactide-*co*-allyl-glycolide) with low allyl content (PLA-*co*-PAGA-L), poly(lactide-*co*-allyl-lactide) with high allyl content (PLA-*co*-PAGA-H), poly(lactide-*co*-glycolide-*co*-allyl-glycolide) (PLGA-*co*-PAGA), poly(lactide-*co*-glycolide-*co*-allyl-valerolactone) (PLGA-*co*-PAVL), copolymers. **c)** The relative degree of polymerization (DP) of lactide (LA), glycolide (GA), allyl-glycolide (AGA) and allyl-valerolactone (AVL) obtained by integration of characteristic peaks in ^1^H NMR spectra of the copolymers. Number average molecular weight (M_n_) and polydispersity (PDI) of copolymers determined by GPC with DMF as the mobile phase and poly(methyl methacrylate) standards.

**Fig. 2:**
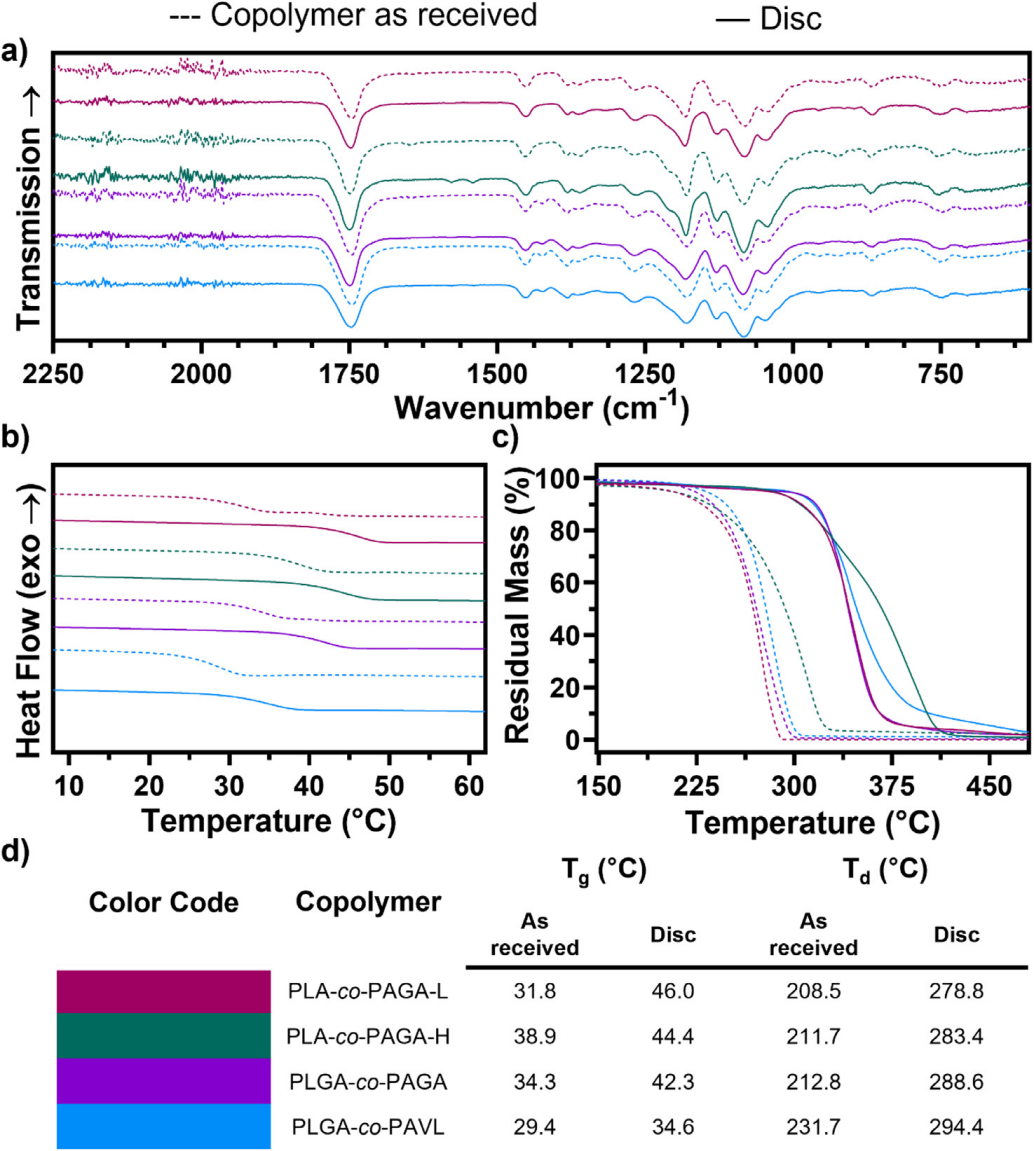
FT-IR spectra **a)**, DSC thermograms **b)**, and TGA traces **c)** of poly(lactide-*co*-allyl-glycolide) with low allyl content (PLA-*co*-PAGA-L), poly(lactide-*co*-allyl-glycolide) with high allyl content (PLA-*co*-PAGA-H), poly(lactide-*co*-glycolide-*co*-allyl-glycolide) (PLGA-*co*-PAGA), poly(lactide-*co*-glycolide-*co*-allyl-valerolactone) (PLGA-*co*-PAVL) copolymers (dashed traces) and corresponding cross-linked discs (solid traces). **d)** Tabulated results of thermal characterization of copolymers and discs showing the glass transition temperature (T_g_) and degradation temperature (T_d_).

**Fig. 3:**
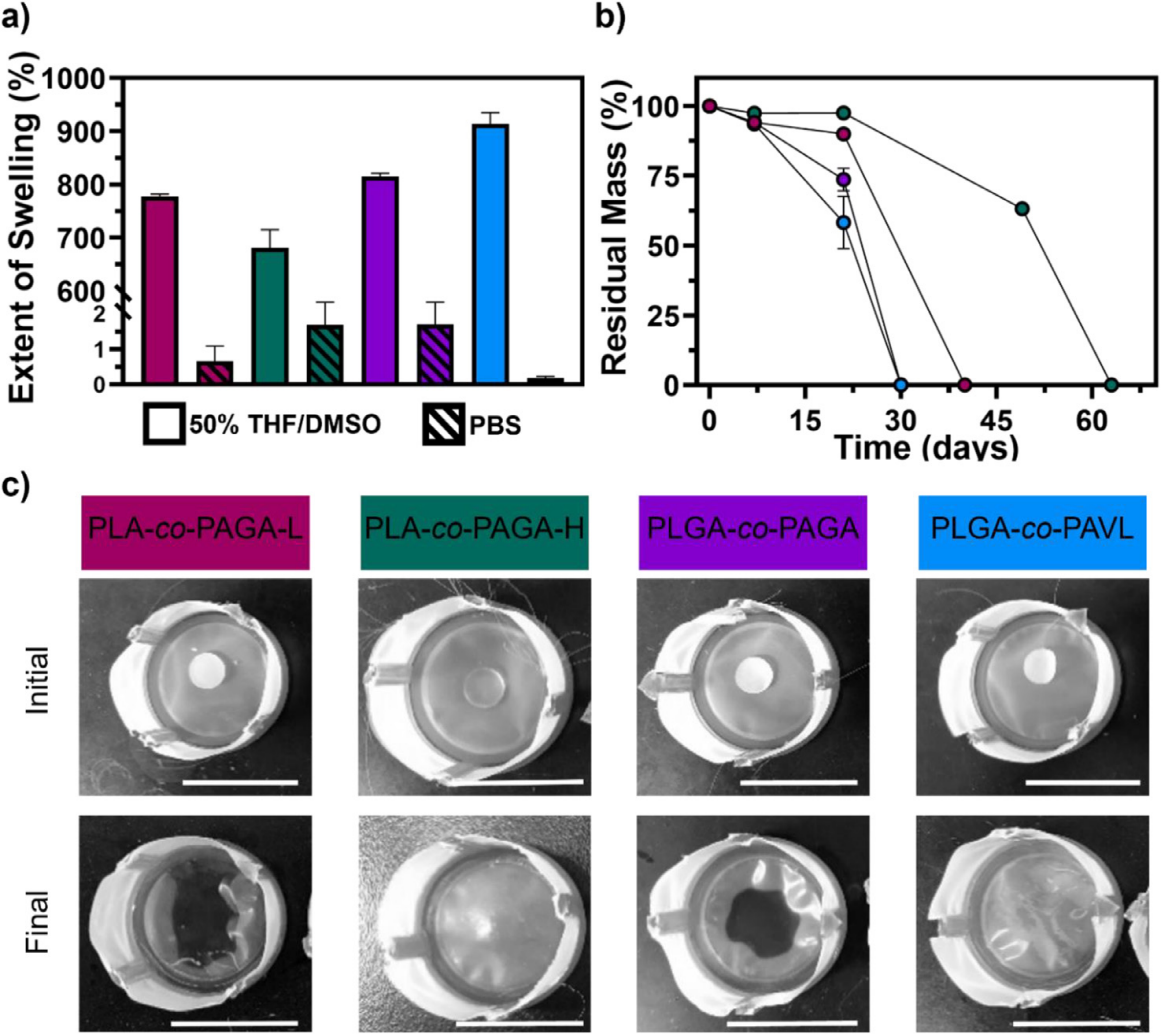
**a)** Extent of swelling of poly(lactide-*co*-allyl-glycolide) with low allyl content (PLA-*co*-PAGA-L), poly(lactide-*co*-allyl-glycolide) with high allyl content (PLA-*co*-PAGA-H), poly(lactide-*co*-glycolide-*co*-allyl-glycolide) (PLGA-*co*-PAGA), poly(lactide-*co*-glycolide-*co*-allyl-valerolactone) (PLGA-*co*-PAVL) discs in a mixture of 50 % (v/v) THF/DMSO at room temperature (solid bars) and in PBS (pH 7.45) at 37.5 °C (striped bars). **b)** Average residual mass of discs in PBS (pH 7.45) at 37.5 °C over time (*n* = 3 ± σ) with **c)** representative images of the discs at the initial and terminal timepoints, the white scale bar shown is 50 mm.

**Fig. 4:**
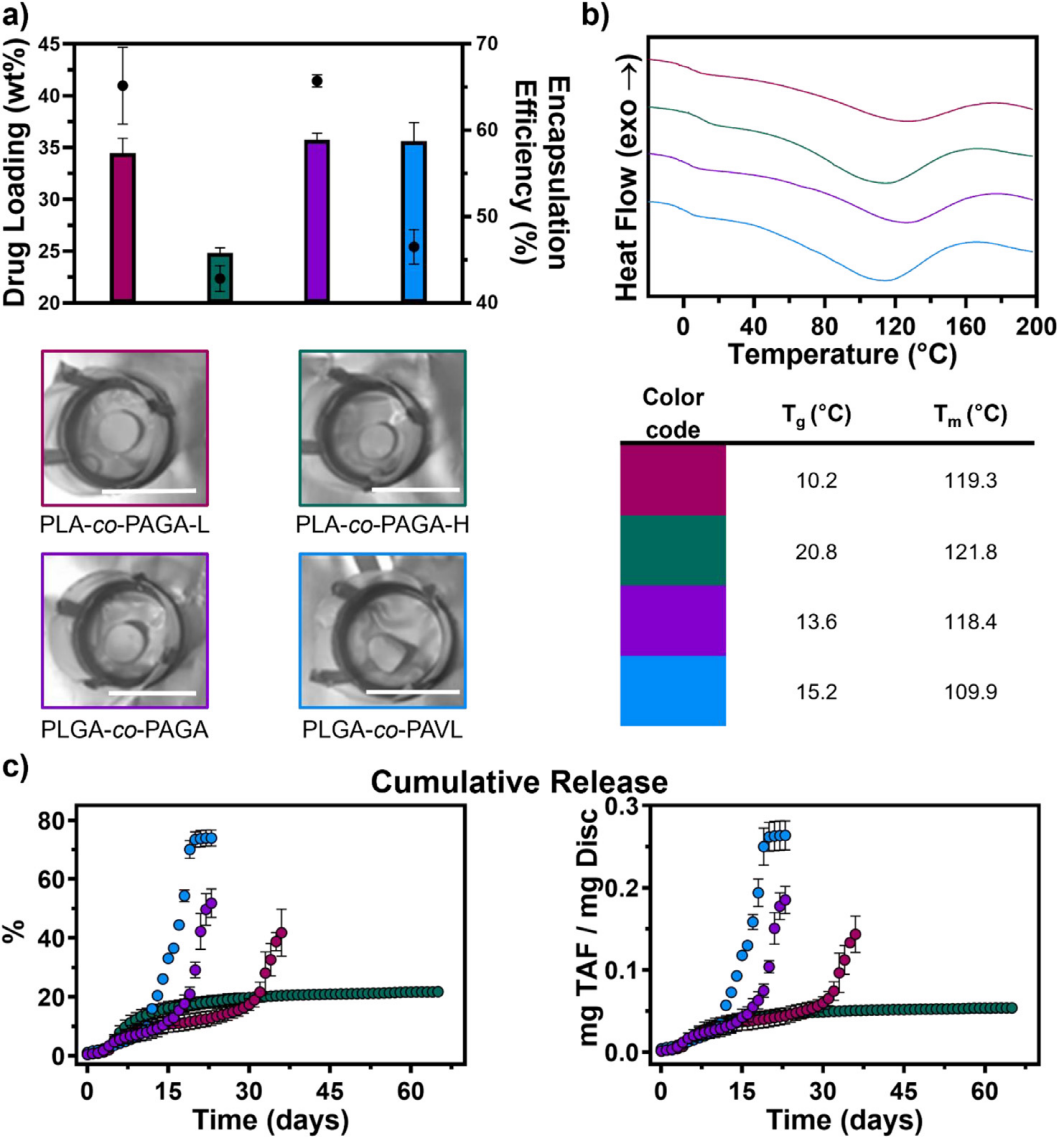
**a)** Average drug loading (solid bars) and encapsulation efficiency (black circles) of tenofovir alafenamide (TAF) in poly(lactide-*co*-allyl-glycolide) with low allyl content (PLA-*co*-PAGA-L), poly(lactide-*co*-allyl-glycolide) with high allyl content (PLA-*co*-PAGA-H), poly(lactide-*co*-glycolide-*co*-allyl-glycolide) (PLGA-*co*-PAGA), poly(lactide-*co*-glycolide-*co*-allyl-valerolactone) (PLGA-*co*-PAVL) discs (*n* = 3 ± σ) with representatives images of the discs shown below (white scale bars are 50 mm). **b)** DSC thermograms of TAF-loaded discs with tabulated glass transition (T_g_) and melting (T_m_) temperatures. **c)** Average *in vitro* release of TAF from the discs in PBS (pH 7.45) at 37 °C (*n* = 3 ± σ).

The data herein builds on a publication evaluating a series of cross-linked polyester disc shaped implants loaded with paclitaxel [9]. In this work, we characterized the properties (*e.g.*, molecular weight and allyl content) of several allyl functionalized polyesters: poly(lactide-*co*-allyl-glycolide) with low allyl content (PLA-*co*-PAGA-L), poly(lactide-*co*-allyl-glycolide) with high allyl content (PLA-*co*-PAGA-H), poly(lactide-*co*-glycolide-*co*-allyl-glycolide) (PLGA-*co*-PAGA), poly(lactide-*co*-glycolide-*co*-allyl-valerolactone) (PLGA-*co*-PAVL), and well as the properties of cross-linked disc-shaped implants prepared from these materials (Fig. 1, Fig. 2, Fig. 3). The implants were loaded with the anti-viral drug TAF and further evaluated including the *in vitro* release (Fig. 4). The dataset used to create Fig. 1, Fig. 2, Fig. 3, Fig. 4 is publicly available (Mendeley Data, [10]) as an excel file with spreadsheets corresponding to each panel within the presented figures.

### 3.1 Copolymer characterization

PLA-*co*-PAGA-L, PLA-*co*-PALA-H, PLGA-*co*-PAGA, and PLGA-*co*-PAVL copolymers were characterized by ^1^H NMR and GPC as received (Fig. 1). The relative allyl degree of polymerization (DP) was 7, 12, 6, 4 % for PLA-*co*-PAGA-L, PLA-*co*-PAGA-H PLGA-*co*-PAGA, and PLGA-*co*-PAVL copolymers, respectively (Fig. 1**a,c**). The number average molecular weight (M_n_) was 9.5, 13.5, 11.0, and 10.2 kg/mol and polydispersity was 1.58, 2.03, 1.99, and 1.40 for PLA-*co*-PAGA-L, PLA-*co*-PAGA-H, PLGA-*co*-PAGA, and PLGA-*co*-PAVL copolymers, respectively (Fig. 1**b,c**).

### 3.2 Spectral and thermal properties of the cross-linked discs

The cross-linked discs were characterized using Fourier transform infrared spectroscopy (FT-IR) (Fig. 2**a**), differential scanning calorimetry (DSC) (Fig. 2**b**), a thermal gravimetry analyzer (TGA) (Fig. 2**c**) and compared to copolymers as received. The three copolymers with the highest relative allyl DP (*i.e.*, PLA-*co*-PAGA-L, PLA-*co*-PAGA-H, PLGA-*co*-PAGA,) show peaks at 1641 cm^−1^ which are not apparent in the corresponding cross-linked discs. Consistent with previous literature [9], the glass transition temperature (T_g_) and degradation temperature (T_d_) of the cross-linked discs increased compared to the copolymers as received (Fig. 2**d**).

### 3.3 Swelling and *in vitro* stability

The swelling of the discs in organic solvent was inversely correlated with the allyl content of the corresponding copolymers and was much higher compared to the swelling in aqueous media (Fig. 3**a**). The *in vitro* stability of the discs was assessed by examining changes in mass as a function of time in PBS showing the stability was proportional to the ally content of the copolymers (Fig. 3**b**). Over time, the disintegration of the discs was visually evident (Fig. 3**c**).

### 3.4 Loading and *in vitro* release

Following loading, the TAF content was 34.5 ± 1.4, 24.8 ± 0.5, 35.8 ± 0.7, and 35.6 ± 1.8 wt% for the PLA-*co*-PAGA-L, PLA-*co*-PAGA-H, PLGA-*co*-PAGA and PLGA-*co*-PAVL discs, respectively (Fig. 4**a**). Representative images show that the TAF-loaded discs were more opaque (Fig. 4**a**) compared to the non-drug loaded discs (Fig. 3**c**). Thermal analysis of the TAF-loaded discs showed a decrease in the T_g_ (Fig. 4**b**) compared to the non-drug loaded discs by ∼15–35 °C (Fig. 2**b,d**). Additionally, a broad melting peak is observed for the TAF-loaded discs at ∼110–120 °C (Fig. 4**b**).

The *in vitro* release of TAF lasted from 3 to over 9 weeks (Fig. 4**c**). For the PLA-*co*-PAGA-L, PLGA-*co*-PAGA, PLGA-*co*-PAVL discs, the release of TAF demonstrated a biphasic profile consisting of an initial slow release (16.3 ± 1.9 %, 12.8 ± 2.6 % and 9.9 ± 0.5 % of cumulative release until 29, 16 and 11 days, respectively) followed by a rapid release (up to 41.8 ± 8.0 %, 51.8 ± 4.8 % and 70.1 ± 3.0 % for 30–36, 17–23 and 12–19 days, respectively) at the later stages. Following the rapid release drug release slowed for 2–3 days followed by cessation of TAF release. The PLA-*co*-PAGA-H discs showed a monophasic release profile with sustained TAF release with minimal burst (1.2 ± 0.5 % in the first day) over 65 days.

## 4 Experimental Design, Materials and Methods

### 4.1 Materials

Poly(lactic-*co*-allyl-glycolic acid) with low allyl content (PLA-*co*-PAGA-L) (Lot #: 180326-AHT-A132)), poly(lactic-*co*-allyl-glycolic acid) with high allyl content (PLA-*co*-PAGA-H) (Lot #: AI072-40430-JSG-C), poly(lactic-*co*-glycolic-*co*-allyl-glycolic acid) (PLGA-*co*-PALA) (Lot #: 180330-SLG-A132), and poly(lactic-*co*-glycolic acid-*co*-allyl-δ-valerolactone) (PLGA-*co*-PAVL) (Lot #: 180731-SLG-B132) were provided by Pendant Biosciences (Toronto, CA) and synthesized by Akina (IN, USA). 2,2-Dimethoxy-2-phenylacetophenone (DMPA, 99 %), 1,6-hexanedithiol (HDT, 96 %), trifluoroacetic acid (TFA, HPLC grade), lithium bromide (LiBr), and dimethyl sulfoxide (DMSO, anhydrous 99.9 %) were purchased from Sigma Aldrich. Tenofovir alafenamide free base (TAF) was provided by Pendant Biosciences. Phosphate buffered saline (PBS, pH 7.45) was purchased from Life Science Corporation (Grand Island, NY). Acetonitrile (ACN, HPLC grade), dichloromethane (DCM, HPLC grade), dimethylformamide (DMF, HPLC grade) tetrahydrofuran (THF, reagent grade) and acetone (reagent grade) were purchased from Caledon Laboratories Ltd. (Georgetown, ON).

### 4.2 Instrumentation

#### 4.2.1 Nuclear magnetic resonance analysis

^1^H NMR spectra of the copolymers as received were recorded using a Bruker AMX 400 spectrometer. The CDCl_3_ singlet at 7.26 ppm was selected as the reference standard. The relative degree of polymerization of the copolymers was determined by the integration ratio of characteristic peaks in the NMR spectra. For the PLA-*co*-PAGA-L and PLA-*co*-PAGA-H copolymers; peaks at 5.9 – 5.6 and 1.6 – 1.5 ppm which correspond to methanylylidene (-C*H*=CH_2_) and lactide methyl groups (-C=OCHC*H_3_*O-), respectively were used. For the PLGA-*co*-PAGA PLGA-*co*-PAVL copolymers; peaks at 5.9 – 5.6, 4.9 – 4.5 and 1.6 – 1.5 ppm were used corresponding to methanylylidene (-C*H*=CH_2_), glycolide methylene (-C=OC*H*_2_O-) and lactide methyl (-C=OCHC*H*_3_O-) groups, respectively.

#### 4.2.2 Gel-permeation chromatography

An Agilent GPC was equipped with a 1260 Infinity isocratic pump and a RI detector. Two Agilent columns (PLgel mixed-D and mixed-C) were used with DMF containing 0.1 mol% LiBr at 50 °C at a flow rate of 1.0 mL/min. Linear poly(methyl methacrylate) standards from Fluka were used for calibration. Aliquots of the copolymers as received were dissolved in DMF/LiBr. The clear solutions were filtered using a 0.22 μm PTFE filter to remove any insoluble species. One drop of anisole was added as a flow rate marker.

#### 4.2.3 Differential scanning calorimetry

Thermal properties including the glass transition temperature (T_g_) of copolymers and their cross-linked discs were measured with a TA Instruments Q100 DSC. Samples were dried under vacuum for 24 h at room temperature to remove residual solvents. Temperature range was from −80 to 180 °C with heating and cooling cycles conducted at a rate of 10 °C/min (cycles: cool to −80 °C, heat up to 180 °C (1^st^ run), cool to −70 °C, heat up to 180 °C (2^nd^ run), and cool to 25 °C).

#### 4.2.4 Thermo-gravimetric analysis

TGA measurements were carried out using a TA instruments Q50 analyzer. Dried pieces of discs (10–15 mg) were placed in a platinum pan and heated from 25 to 600 °C at a heating rate of 20 °C/min under nitrogen flow.

#### 4.2.5 FT-IR

FT-IR spectra of all the samples were recorded by a Nicolet FT-IR spectrometer attached with an attenuated total reflectance (ATR) accessory. All spectra were recorded with 32 scans at room temperature in the range of 600–4000 cm^−1^ at 1 cm^−1^ resolution.

### 4.3 Formulation of cross-linked discs

Disc-shaped implants were synthesized as previously described [9]. In brief, a reactive solution was prepared by dissolving 250 mg of each copolymer, 0.5 allyl molar equivalents of HDT and 1 allyl molar equivalents of DMPA in 1 mL of DMSO. The solution was injected into a glass mold (20 mm × 30 mm × 3 mm) and exposed to UV (365 nm) for 20 min. The cross-linked polymer film was removed from the mold and washed using acetone to remove residual reagents rinsed with water and dried at room temperature under vacuum for 2 days. A 6 mm punch (Interga Miltex NJ, USA) was used to prepare the discs.

### 4.4 Swelling characterization

The extent of swelling of the cross-linked discs was evaluated in organic solvent and aqueous media. Cross-linked discs, as described above (approximately 25–30 mg), were placed in a mixture of THF/DMSO (50/50 % v/v) at room temperature for 8 hrs. They were also placed in the release media (PBS, pH 7.45) at 37.5 °C for a week. The extent of swelling was calculated as the ratio of the weight of the swollen discs to the initial weight for the discs.

### 4.5 *In vitro* stability

For *in vitro* degradation, the discs (25–30 mg) were immersed in PBS (10 mL, pH = 7.45) and placed in an incubator at a temperature of 37.5 °C, and the media was replaced with fresh PBS weekly. Samples were weighed at certain time points (*i.e.*, 1 week, 3 weeks, 5 weeks, 7 weeks) after they were washed with an excess amount of deionized water three times and dried by lyophilization for two days. The weight change was calculated using the following equation:

$$Weight\;change\;\left(\%\right)=\left(\frac{Weight\;of\;remaining\;disc}{Weight\;of\;initial\;disc}\right)\times \;100\%$$

The weight of discs was denoted as zero once they disintegrated into small pieces.

### 4.6 Drug loading

Each disc was weighed (25–30 mg) and placed in a drug solution that consisted of TAF (25 mg) and 0.5 mL of a mixture of THF and DMSO (50/50 % v/v) to allow for post-loading through a swelling and equilibration method for 8 h. The disc was then transferred to a new vial and lyophilized overnight. Following, the disc was briefly rinsed using 10 mL of fresh THF twice to remove drug present at the surface of the disc and then lyophilized for two days. The drug loading and encapsulation efficiency (EE) were determined using the following equations:

$$Drug\;Loading\;\left(\%\right)=\frac{Mass\;of\;TAF\;loaded\;disc-Mass\;of\;initial\;disc}{Mass\;of\;TAF\;loaded\;disc}\times \;100\%$$

$$EE\left(\%\right)=\frac{Mass\;of\;TAF\;loaded\;disc-Mass\;of\;initial\;disc}{Mass\;of\;TAF\;used\;for\;loading}\times \;100\%$$

### 4.7 HPLC analysis

TAF samples were analyzed by reverse phase HPLC using an Agilent 1200 HPLC system with Agilent Chem Station software, an XBridge BEH C18 column (3.5 µm, 4.6 × 50 mm + guard column) (Water Corp., Milford, MA) and UV detection. The mobile phase consisted of 30/70 ACN/water (with 0.1 % of trifluoroacetic acid) at a flow rate of 1 mL/min and UV detection at 260 nm. TAF standards were dissolved in 50 mM sodium acetate buffer (pH 4.5) ranging from 0.5 to 200 μg/mL. The runtime was 3 min and TAF eluted at 1.9 min.

### 4.8 *In vitro* release

TAF loaded discs were placed in a cell strainer and immersed in the release media (10 mL of PBS, pH = 7.45) in a 30 mL beaker which was sealed and incubated at 37.5 °C under stirring. Aliquots (1 mL) were taken from the release media at certain time points and samples were stored at −20 °C until HPLC analysis. The remaining release media was discarded and replaced by fresh media (10 mL) every time a sample was removed for drug analysis. The release was determined to cease when the concentrations fell below the limit of detection for the HPLC assay (*i.e.*, 0.5 µg/mL).

## Limitations

The scope of the dataset is limited to *in vitro* characterization of the implants. It is known that *in vitro* properties of implants don't always translate to *in vivo* performance. Furthermore, these studies were time-and-resource intensive to conduct which limited the number of materials that were characterized.

## Ethics Statement

This work does not involve human studies, animal studies, or data collected from social media platforms. All Authors have read and followed the ethical requirements for publication in Data in Brief.

## CRediT authorship contribution statement

**Sungmin Jung:** Conceptualization, Methodology, Investigation, Visualization, Writing – original draft, Writing – review & editing. **Jack Bufton:** Methodology, Visualization, Investigation, Writing – original draft, Writing – review & editing. **Zeqing Bao:** Investigation, Writing – review & editing. **Wonjoon Cho:** Methodology, Investigation. **Dean Aguiar:** Project administration, Funding acquisition. **Christine Allen:** Conceptualization, Project administration, Writing – review & editing, Supervision, Funding acquisition.

## Data Availability

Mendeley DataPhysico-chemical characterization of a panel of cross-linked polyester implants (Original data).
